# Uracil moieties in *Plasmodium falciparum* genomic DNA

**DOI:** 10.1002/2211-5463.12458

**Published:** 2018-09-29

**Authors:** Petra Molnár, Lívia Marton, Richard Izrael, Hajnalka L. Pálinkás, Beáta G. Vértessy

**Affiliations:** ^1^ Research Centre for Natural Sciences Institute of Enzymology BME‐MTA Malaria Research Laboratory Hungarian Academy of Sciences Budapest Hungary; ^2^ Department of Applied Biotechnology and Food Science Budapest University of Technology and Economics Budapest Hungary; ^3^ Doctoral School of Multidisciplinary Medical Science University of Szeged Szeged Hungary

**Keywords:** base‐excision repair, DNA damage and repair, malaria, Uracil‐DNA repair, *Plasmodium falciparum*, Uracil‐DNA detection

## Abstract

*Plasmodium falciparum* parasites undergo multiple genome duplication events during their development. Within the intraerythrocytic stages, parasites encounter an oxidative environment and DNA synthesis necessarily proceeds under these circumstances. In addition to these conditions, the extreme AT bias of the *P. falciparum* genome poses further constraints for DNA synthesis. Taken together, these circumstances may allow appearance of damaged bases in the *Plasmodium* DNA. Here, we focus on uracil that may arise in DNA either via oxidative deamination or thymine‐replacing incorporation. We determine the level of uracil at the ring, trophozoite, and schizont intraerythrocytic stages and evaluate the base‐excision repair potential of *P. falciparum* to deal with uracil‐DNA repair. We find approximately 7–10 uracil per million bases in the different parasite stages. This level is considerably higher than found in other wild‐type organisms from bacteria to mammalian species. Based on a systematic assessment of *P. falciparum* genome and transcriptome databases, we conclude that uracil‐DNA repair relies on one single uracil‐DNA glycosylase and proceeds through the long‐patch base‐excision repair route. Although potentially efficient, the repair route still leaves considerable level of uracils in parasite DNA, which may contribute to mutation rates in *P. falciparum*.

**Abbreviations**
3meA: 3‐methyladenine
3meG: 3‐methylguanine
5‐FU: 5‐fluorouracil
5‐hC: 5‐hydroxycytosine
5‐hmU: 5‐hydroxymethyluracil
5‐hU: 5‐hydroxyuracil
7meG: 7‐methylguanine
8‐oxoG: 7,8‐dihydro‐8‐oxoguanine
AP lyase: apurinic/apyrimidinic lyase
APE1: apurinic/apyrimidinic endonuclease 1
BER: base‐excision repair
DHU: dihydrouracil
dut‐ ung‐: deoxyuridine‐triphosphatase/uracil‐DNA glycosylase double knockout
DUT: deoxyuridine‐triphosphatase
FapyA or G: formamidopyrimidine lesions
FEN1: flap endonuclease 1
Hx: hypoxanthine
LIG 1/3: DNA ligase 1/3
MBMD 4: methyl–CpG‐binding domain protein 4
MPG: DNA‐3‐methyladenine glycosylase
MUTYH: mutY homolog
NEIL 1/2/3: endonuclease VIII like enzyme 1/2/3
NTHL1: endonuclease III‐like protein 1
OGG 1: 8‐oxoguanine glycosylase 1
PCNA: proliferating cell nuclear antigen
PNKP: polynucleotide kinase/phosphatase
Pol β/δ/ε: DNA polymerase β/δ/ε
RFC: replication factor C
SMUG 1: single‐strand selective monofunctional uracil‐DNA glycosylase
TDG: thymine‐DNA glycosylase
Tg: thymine glycol
UNG: uracil‐DNA glycosylase
XRCC1: X‐ray repair cross‐complementing protein 1

Malaria is a major health threat affecting large regions globally, resulting in the death of ~450 000 people annually 1. The parasite's capability of adaptation is a major hindering factor in the way of eliminating the disease, mostly represented by the growing resistance of parasites against antimalarials 1. The causative agents of malaria belong to the *Plasmodium* genus. Among the five human parasites, *Plasmodium falciparum* (*P. falciparum*) presents an exceptional biomedical challenge being responsible for the most serious infections and most of the lethal cases 2.

The life cycle of *P. falciparum* is intriguingly complex (Fig. 1). The parasites undergo multiple DNA replications at several developmental stages in their vector (*Anopheles* mosquito) and host (human liver and bloodstream). The sexual phase of development occurs in the female *Anopheles* mosquito. The only meiotic division takes place when the zygote, originating from the fusion of microgametes and macrogametes inside the mosquito, evolves into ookinetes. These will then develop into multinuclear oocysts, wherein mitotic sporogenesis results in the formation of numerous sporozoites. After the mosquito bites a human host, sporozoites invade the liver and undergo at least a dozen rounds of mitosis to produce tens of thousands of haploid merozoites 3. These start the intraerythrocytic cycle by the invasion of red blood cells. The importance of this cycle is emphasized by the fact that about two‐thirds of the genes of a murine *Plasmodium* have been shown to be necessary for the blood stage growth of parasites 4. First, they develop into rings, followed by trophozoites. At this stage, parasites enter the G1 phase, and start to prepare for DNA replication. The S phase starts around 30 h after erythrocyte invasion, when parasites are in the late trophozoite stage. The replication in the parasite is asynchronous and produces up to about 24n sister chromatids. Replication ends around 44 h postinvasion, after which each genome is packed into daughter merozoites (schizont form) 3, 5. Merozoites may exit the continuous intraerythrocytic cycle by differentiating into male or female gametocytes. These sexual forms, consumed by the mosquito, evolve into micro‐ or macrogametocytes. Microgametocytes undergo three mitotic cycles and form eight exflagellated microgametes 3.

**Figure 1:**
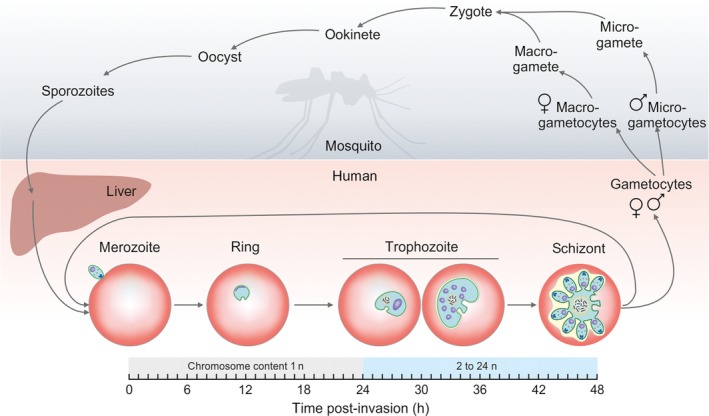
The life cycle of *Plasmodium falciparum*. Stages within the mosquito vector and inside the human host are on light gray or coral background, respectively. Developmental stages of the intraerythrocytic cycle are represented by graphical illustrations, and changes in chromosome content in these stages are also indicated on a schematic horizontal axis.

Importantly, DNA replication cycles within the intraerythrocytic stages proceed in an environment rich in oxidative conditions. Especially, free heme and iron during the hemozoin formation process pose notable oxidative stress, which may result in DNA modifications (e.g., oxidative cytosine deamination leading to uracil and other oxidative processes). The specific composition of genomic DNA in *Plasmodia* may also facilitate the appearance of uracil in the DNA. Members of the *Plasmodium* genus possess the most AT‐rich genome sequenced so far, including *P. falciparum*, having namely ~80% AT content in the exonic regions and ~90% in the intronic regions 6, 7. Comparing to other organisms, like the host, *Homo sapiens*, where the average AT content is 58.9%, and to other eukaryotic pathogens, namely *Toxoplasma gondii* and *Trypanosoma brucei*, having 47.7% and 53.2% AT, respectively, the base composition of the *P. falciparum* genome is indeed extraordinary 8. A mutation bias has recently been pointed out in *P. falciparum*, showing an increased occurrence of GC→AT substitutions, which could promote the AT‐rich genome structure 8. The AT richness of the genome may increase the possibility of uracil content, as huge amounts of thymidines are incorporated, giving more chance for the polymerase to mistake thymidines with uridines as compared to a genome with lower levels of AT content. We therefore wished to determine uracil levels in genomic DNA of *P. falciparum* during the intraerythrocytic stages.

A dot‐blot‐based uracil detection method has recently been developed in our laboratory, which provides a robust and straightforward possibility for sensitive and quantitative detection of uracil‐DNA levels 9. The basis of detection is an engineered catalytically inactive uracil‐DNA glycosylase (UNG), which is capable of recognizing and binding to uracils incorporated in DNA sequences. The uracil sensor UNG‐construct can be equipped with diverse tags for ease of detection via antibodies using the dot‐blot method. The sensitivity of the method is equivalent to that of MS‐based methods, providing a limit in femtomolar concentrations 9.

In the present work, we analyzed the uracil content of genomic DNA from three different intraerythrocytic developmental stages of *P. falciparum* 3D7 parasites, namely the ring, trophozoite, and schizont stages. The quantification of uracil moieties was performed by the aforementioned dot‐blot‐based uracil detection method 9. To assess the potential efficiency of uracil‐DNA repair, we compared the existing orthologues of mammalian base‐excision repair enzymes to those present in the parasite based on genome databases of *H. sapiens* and *P. falciparum*. We also analyzed transcriptome databases of the intraerythrocytic parasite stages with regard to expression level of base‐excision repair enzymes.

## Materials and methods

### Maintenance of parasite cultures

*Plasmodium falciparum* 3D7 parasites were obtained from the University of Montpellier. Continuous cultures were maintained in human 0+ erythrocytes. Parasites were grown at 5% hematocrit (HCT) in complete RPMI medium [incomplete RPMI 1640 (w l‐glutamine, w Hepes, w NaHCO_3_)] (Lonza, Basel, Switzerland) supplemented with 50 mg·L^−1^ gentamicin (VWR Chemicals, Radnor, PA, USA), 37 µm hypoxanthine (Alfa Aesar, Haverhill, MA, USA), and 1.25 g·L^−1^ Albumax I (Gibco from Thermo Fisher Scientific, Waltham, MA, USA). Cultures were kept at 37 °C in a laboratory incubator gassed with 5% O_2_, 5% CO_2_, and 90% N_2_. Cultures were synchronized by sorbitol at the young ring stage, and Percoll treatment at the schizont stage matured from sorbitol‐treated rings, as described elsewhere 10, 11. Trophozoites were obtained from synchronized cultures, after parasites reached the early trophozoite stage.

### Genomic DNA isolation

Synchronized parasites of different developmental stages were collected from two biologically independent cultures (i.e., biological replicates), and lysed as described elsewhere 12. Briefly, red blood cells were lysed in 5% saponin (Sigma‐Aldrich, St. Louis, MO, USA) in PBS, then incubated at 37 °C for 3 h for parasite lysis in a lysis solution (pH = 7.5) of the following composition: 40 mm Tris/HCl; 80 mm EDTA; 2% SDS; 0.1 mg·mL^−1^ proteinase K. After treatment, genomic DNA was purified using the QuickDNA Miniprep Plus kit obtained from Zymo Research (Irvine, CA, USA).

### Dot‐blot measurement and analysis

Dot‐blot measurements were carried out in four independent replicates using samples from the *P. falciparum* developmental stages, namely rings, trophozoites, and schizonts, as described elsewhere 9. Briefly, genomic DNA isolated from CJ236 *Escherichia coli* strain [*dut*−*,ung*−] served as a uracil standard, applied in 15 ng diluted into 1 μg of ultrapure salmon sperm DNA. The standard was diluted in a 1/2; dilution series. The two‐third serial dilutions for *P. falciparum* samples started with 1 μg of DNA. Samples were spotted onto a prewetted positively charged nylon membrane (Amersham Hybond‐Ny+; GE Healthcare, Little Chalfont, UK) using a vacuum‐driven microfiltration apparatus (Bio‐Dot, Bio‐Rad, Hercules, CA, USA). The DNA was immobilized, and the membrane was blocked and incubated with the detector construct of UNG. After several washing steps, first primary, then secondary antibodies were applied. Immunoreactive bands were visualized by enhanced chemiluminescence reagent (Western Chemiluminescent HRP substrate from Merck Millipore, Burlington, MA, USA), and images were captured by a Bio‐Rad ChemiDocTM MPImaging system. Densitometry was performed using imagej 1.48p software (National Institutes of Health, Bethesda, MD, USA). The number of deoxyuridine nucleotides was calculated as described elsewhere 9. Calibration curve from the dilution of the standard was fitted with a polynomial with second order that provided a fit with *R* ^2 ^≥ 0.98. The number of uracil per million bases in the ‘unknown’ genomic DNA was determined by interpolating their normalized intensities in the calibration plot based on the amount of DNA applied.

### Statistical analysis

Statistical analysis was carried out by originpro 8.6 (OriginLab, Northampton, MA, USA) using one‐way ANOVA test when samples passed homogeneity of variance test (Levene's test) and normal distribution tests (Kolmogorov–Smirnov test). Differences were considered statistically significant at *P < *0.05.

### Transcriptome analysis

Transcriptome analysis was carried out using the Transcriptomics function of the Plasmodb database. The gene expression level of each protein was estimated by RNA‐Seq data for intraerythrocytic stages 13, 14. Raw data were plotted for four stages: ring, early and late trophozoite, and schizont.

## Results and Discussion

**Figure 2:**
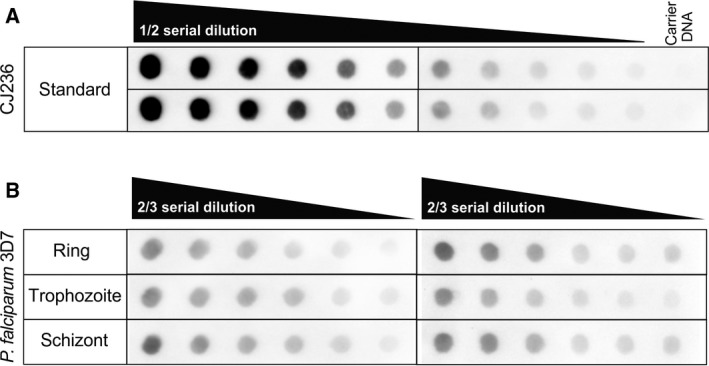
Dot‐blot assays for measuring genomic uracil levels of the different developmental stages of *Plasmodium falciparum* parasites. (A) CJ236 [*dut*−*, ung*−] *Escherichia coli* genomic DNA was used as standard for the dot‐blot assay. (B) Representative dot‐blot images of the measurement of the quantity of genomic uracil in *P. falciparum* ring, trophozoite, and schizont samples.

We measured the amount of uracil moieties/million bases of DNA samples extracted from the three intraerythrocytic developmental stages of the *P. falciparum* parasites using a recently developed dot‐blot‐based detection method 9. Parasite cultures were synchronized, and cultures of ring, trophozoite, and schizont were collected for the isolation of genomic DNA. Two representative dot‐blot images are shown in Fig. 2. The measured amount of uracil moieties in our samples could be fitted to the linear range of the standard dilution series (Fig. 2).

The uracil content of ring, trophozoite, and schizont stage parasites were determined to be 9.6 ± 2.8, 6.7 ± 2.4, and 7.6 ± 3.8 uracil per million bases, respectively. One‐way ANOVA statistical analysis revealed that the uracil contents of the measured erythrocytic stages under these experimental conditions do not differ significantly from each other (*P* = 0.618).

It is of interest to note that these uracil‐DNA levels are significantly higher than those observed in other samples from different wild‐type organisms. The level of uracil moieties in genomic DNA has been assessed by various methods in numerous organisms so far 9, 15, 16 The general conclusion from these studies agrees that wild‐type organisms from bacteria to mammals, as well as normal cell lines, show low levels of uracil in DNA in the range of 0.1–1 uracil per million bases, or even lower 16, 17. An interesting exception was found in *Drosophila* S2 cells, where the uracil‐DNA content was reported to be around 15–16 uracil per million bases 9. This considerable high genomic uracil level is, however, probably directly related to the lack of the most efficient uracil‐DNA glycosylase enzyme (UNG protein) from the Drosophila genome 18. Organisms under genotoxic stress or engineered to lack uracil‐DNA glycosylases also present increased genomic uracil levels 9, 15, 16, 17. To discuss our results, it was therefore of immediate interest to investigate whether the approx. 7–10 uracil per million bases levels in the *P. falciparum* genomic DNA samples may be related to a limited set of repair enzymes in the parasite.

The DNA repair route to remove uracil from DNA relies on the base‐excision repair (BER) pathway 19. We therefore systematically compared the relevant set of proteins encoded in mammalian species vs *P. falciparum*. For the initial search of the related *P. falciparum* BER enzyme set, the KEGG pathway database 20 was used with manual curation and verification of the hits. In each case, we also performed a sequence alignment to decide whether the orthologues are truly relevant and include the functionally important residues. Whenever available, published studies on the specific proteins were also consulted. Results are shown in Table 1 and identify two interesting limitations of the BER protein set in *P. falciparum*.

**Table 1:** Comparison of the mammalian and *Plasmodium falciparum* BER protein sets and their involvement in short‐patch versus long‐patch BER (cf ‘x’ marks). The functionality of DNA glycosylases is defined as mono‐ (M) or bifunctional (B). Question mark in case of DNA polymerase β indicates that a polymerase β‐like enzyme was reported in *P. falciparum*, with an activity related to mammalian Pol β; however, the respective gene is not annotated. All abbreviations are listed in the Abbreviations section of the article

Mammalian 19		Functionality (for glycosylases only)	Substrates	Short‐patch BER	Long‐patch BER	Plasmodium orthologue	UniProt/PlasmoDB ID	Ref.
DNA N‐glycosylase	**UNG1/2**	M	U, 5‐FU, U:A, U:G	x	x	Uracil‐DNA glycosylase	http://www.uniprot.org/uniprot/Q8ILU6/PF14_0148	21
	TDG	M	U:G>T:G	x	x	Not found		
	SMUG1	M	U:G>U:A, 5‐FU, 5‐hmU	x	x	Not found		
	MBD4	M	U:G, T:G	x	x	Not found		
	**NTHL1**	B	Tg, FapyG, 5‐hC, 5‐hU	x	x	Endonuclease III homologue	http://www.uniprot.org/uniprot/C6KSY9/PFF0715c	6
	**OGG1**	M/B	8‐oxoG:C, FapyG	x	x	N‐glycosylase/DNA lyase	http://www.uniprot.org/uniprot/Q8I2Y2/PFI0835c	6
	**MUTYH**	M	A opposite 8‐oxoG	x	x	A/G‐specific adenine glycosylase	http://www.uniprot.org/uniprot/Q8II68/PF11_0306	6
	**MPG**	M	3meA, 7meG, 3meG, Hx	x	x	DNA‐3‐methyladenine glycosylase	http://www.uniprot.org/uniprot/Q8IKG6/PF14_0639	6
	NEIL1	B	Tg, FapyG, FapyA, 8‐oxoG, 5‐hU, DHU	x	x	Not found		
	NEIL2	B	Tg, FapyG, FapyA, 8‐oxoG, 5‐hU, DHU	x	x	Not found		
	NEIL3	M/B	FapyA, FapyG	x	x	Not found		
AP endonuclease	**APE1**			x	x	Apurinic/apyrimidinic endonuclease Apn1	http://www.uniprot.org/uniprot/Q8IE02/PF13_0176	22
	**AP lyase**			x	x			
Polymerase	Pol β			x	x	?	?	23
	**Pol δ**			–	x	DNA polymerase δ	http://www.uniprot.org/uniprot/Q7KQL4/PF10_0165	21, 24
	**Pol ε**			–	x	DNA polymerase ε	http://www.uniprot.org/uniprot/C6KTD8/PFF1470c	6, 25
Flap endonuclease	**FEN1**			–	x	flap endonuclease 1	http://www.uniprot.org/uniprot/Q7K734/PFD0420c	26
DNA ligase	**LIG1**			x	x	DNA ligase I	http://www.uniprot.org/uniprot/Q8IES4/MAL13P1.22	27
	LIG3			x	–	not found		
Factors	**PNKP**			x	–	polynucleotide kinase/phosphatase	http://www.uniprot.org/uniprot/Q8ID74/PF13_0334	28
	XRCC1			x	–	not found		
	**PCNA**			–	x	proliferating cell nuclear antigen	http://www.uniprot.org/uniprot/P61074/PF13_0328	29, 30
						proliferating cell nuclear antigen 2	http://www.uniprot.org/uniprot/Q7KQJ9/PFL1285c	30, 31, 32
	**RFC**			–	x	P‐loop containing nucleoside triphosphate hydrolase	PFA0545c	33, 34

Enzymes written in bold designate the mammalian enzymes with Plasmodium orthologs.

These two limitations relate to, on the one hand, the set of enzymes capable of recognizing and cleaving uracil from DNA, and on the other hand, to the set of proteins required for the short‐patch versus long‐patch BER routes. Uracil‐DNA glycosylases in diverse organisms include at least four enzyme families (UNG, TDG, SMUG, MBD4) 35, 36. The diversity in these enzymes defines their specific roles and different substrate specificities and underlies the high significance of uracil removal from DNA. In *P. falciparum*, however, only one uracil‐DNA glycosylase gene is present: It encodes the archetypical UNG enzyme.

With regard to the second limitation, concerning short‐patch vs long‐patch BER pathways, it has been argued earlier that *P. falciparum* predominantly employs the long‐patch pathway 37. In agreement with this study, several proteins involved in short‐patch BER was not identified in *P. falciparum* (e.g., polymerase β, ligase 3) (cf Table 1). It has to be mentioned that although a protein with polymerase β‐like enzyme activity has been reported in *P. falciparum* 23, its role in short‐patch BER in *P. falciparum* has not been confirmed. As it is responsible for the synthesis of 3‐ to 5‐bp oligonucleotides, it may be involved in long‐patch repair 23. Also, this ‘polymerase β‐like’ protein may have another role in an alternative end‐joining pathway in the parasite 38. No orthologues of the LIG3 and its stabilizing scaffold protein, XRCC1, have been found so far. LIG3 and XRCC1 are responsible for the ligation process in short‐patch BER 19. In summary, the protein set encoded in *P. falciparum* is deficient on short‐patch BER, but all protein orthologues necessary for the long‐patch BER pathway have been clearly identified in the parasite.

**Figure 3:**
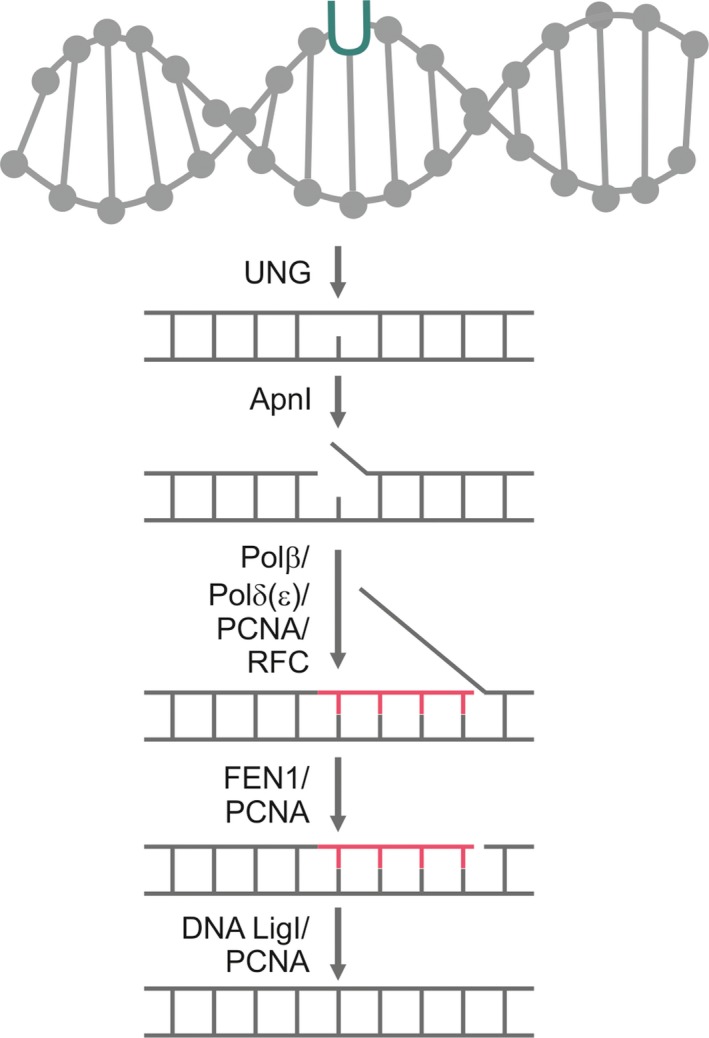
A possible uracil‐DNA repair mechanism of *Plasmodium falciparum* via long‐patch BER, based on the analysis of BER enzyme sets.

Based on these data, a possible route of *P. falciparum* long‐patch BER‐based uracil‐DNA repair mechanism is shown in Fig. 3. The recognition and excision of uracil in the DNA of the parasites are performed by UNG. The next step is DNA strand cleavage by Apn1, which results in the formation of a nick in the DNA backbone. DNA polymerase δ (or ε) binds to the DNA by the help of proliferating cell nuclear antigen (PCNA) and the replication factor C (RFC) orthologue to start the synthesis of ~10 new nucleotides, while removing the downstream 5′ DNA end. The replaced section forms a so‐called flap structure that is still connected to the DNA. It is removed by flap endonuclease 1 (FEN1). The leftover nick is ligated by DNA ligase I 39.

**Figure 4:**
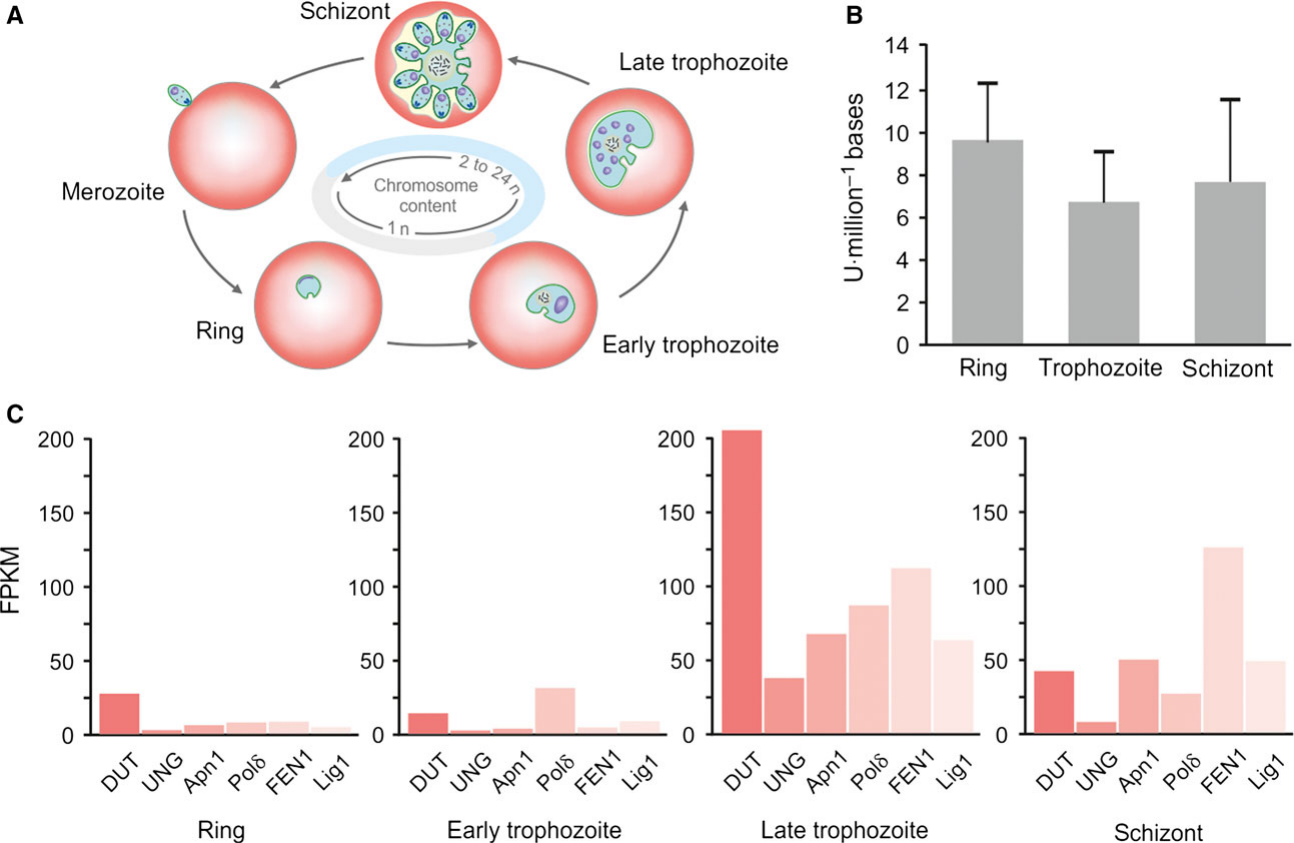
Uracil‐DNA and repair enzymes expression levels in intraerythrocytic *Plasmodium falciparum* stages. (A) Changes in chromosome content in the different stages. (B) The uracil‐DNA levels in the ring, trophozoite, and schizont stage parasites are shown with error bars. (C) Analysis of transcriptomes of the long‐patch BER enzyme set in *P. falciparum* intraerythrocytic developmental stages. FPKM is the transcript levels of fragments per kilobase of exon model per million mapped reads.

It was also of interest to look into the expression profiles of the key enzymes involved in uracil‐DNA repair in the parasite, in relation to the uracil‐DNA levels during the intraerythrocytic stages. In this analysis, we also considered the dUTPase enzyme, which is responsible for cleaving dUTP to prevent uracil incorporation into DNA 36. Based on the analysis of BER enzyme transcriptome levels of the different *P. falciparum* developmental stages, the pathway is initiated in the late trophozoite stage (Fig. 4). This is in good agreement with the DNA metabolism of the parasites, as DNA synthesis starts in the late trophozoite stage followed by DNA packaging into merozoites at the end of the intraerythrocytic cycle. In case of UNG and Polδ, the expression level drops again in schizont stage. Apn1, FEN1, and DNA ligase I remain present after late trophozoite stage as well (Fig. 4C). The expression level of dUTPase, responsible for preventing uracil incorporation, is highly elevated in late trophozoite stage in parallel with the BER enzymes. However, transcriptome analysis data should be evaluated with caution as they may not reflect the efficiency of the enzymes participating in uracil repair.

## Conclusions

We have determined uracil‐DNA levels in different intraerythrocytic stages of *P. falciparum* genomic DNA and found that approx. 7–10 uracil per million bases can be detected. To account for this level, which is significantly higher as compared to other normal wild‐type organisms, the balance between processes leading to uracil presence in DNA and its removal needs to be considered.

**Figure 5:**
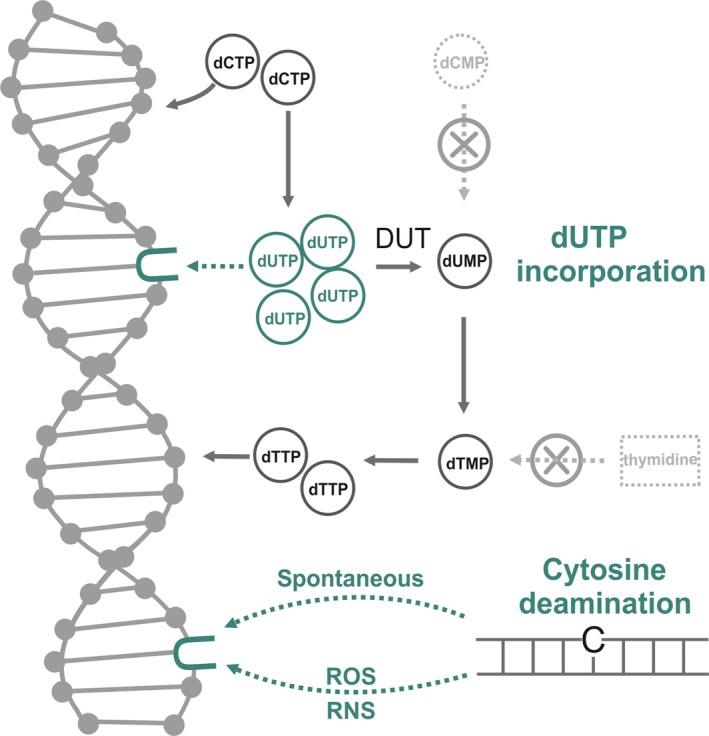
Pathways leading to uracil appearance in DNA. Steps directly resulting in uracil appearance are marked by dark green. Pathways present in mammals but not in Plasmodia are marked by light gray.

There are two mechanisms by which uracil can arise in DNA, as shown in Fig. 5. On the one hand, if cellular dUTP levels are high as compared to dTTP levels, polymerases can readily incorporate dUMP moieties into DNA. The enzyme family of dUTPases are responsible for keeping dUTP levels at a low value to prevent thymine‐replacing incorporations. The significance of this DNA repair function of *P. falciparum* dUTPase is underlined by numerous studies that focus on plasmodial dUTPase inhibition as an important chemotherapeutic strategy against malaria 40, 41, 42.

Thymine‐replacing uracil incorporation into *P. falciparum* genomic DNA may be enhanced by the exceptionally high AT content of the parasite genome. Also, the level of dUTPase expression as suggested by transcriptomic analysis is increased only in the late trophozoite stages, potentially allowing uracil incorporation at earlier stages where DNA replication is initiated. This pathway of uracil incorporation does not result in a stable mutation, but is considered dangerous because high levels of uracil in the DNA can lead to the hyperactivity of the uracil‐repair mechanism, resulting in thymine‐less cell death 36.

Another possibility for uracil to appear in DNA is the oxidative deamination of cytosine, resulting in a G:U pair instead of G:C 36, 43. In the next round of replication, DNA polymerases will incorporate adenosine opposite of U. Without repair, this will lead to the formation of an AT pair, aka a GC→AT substitution. The deamination of cytosine is considered one of the most frequent DNA mutations, with a rate of 100 to 500 U·cell^−1^·day^−1^ 44. In case of *P. falciparum*, the detoxifying process resulting in the formation of hemozoin crystals gives rise to the formation of oxidative agents. The presence of such reactive oxygen and nitrogen species in the parasitized erythrocytic environment can cause the deamination of cytidine in increased frequency, possibly contributing to the GC→AT substitutions 8.

Uracil removal from DNA requires the base‐excision repair process. In mammalian cells, both short‐ and long‐patch BER pathways are present for the repair of base excisions, but the parasites rely only on the long‐patch repair. Moreover, from the different families of uracil‐DNA glycosylases, *P. falciparum* contains only the single UNG protein, further limiting the capacity of parasites to remove uracil from the DNA.

It has been discussed that genomic architecture of *P. falciparum*, containing low complexity regions and repetitive sequences as a consequence of the AT richness, allows high indel mutation rates in coding and noncoding regions. Indel mutations occur 10‐fold more frequently compared to base‐pair substitutions, and this is probably the result of DNA polymerase slippages and unequal crossing over events 8. Possible advantages of high mutation rates include an effect on gene regulation, an extended antigenic variance, a role in drug resistance, and an evolutionary benefit. The mutation of noncoding genes can have an effect on the gene expression, as these regions often have enhancer or repressor roles 8, 45. Probably, the high mutation rates combined with low complexity regions can facilitate adaptive evolution in *P. falciparum* parasites 8. The presence of uracil moieties in the parasite genome may also contribute to mutation rates.

## Author contributions

PM and LM participated in all preparations, experiments, data analysis, figure, and table preparation as well as in writing the study. RI analyzed *P. falciparum* BER enzyme orthologues and expression data. HLP participated in the dot‐blot measurements and the related data analysis. BGV conceived and coordinated the study. The manuscript of this study was reviewed and approved by all authors.

## Conflict of interest

The authors declare no conflict of interest.
